# Enhancing the UV-Light Barrier, Thermal Stability, Tensile Strength, and Antimicrobial Properties of Rice Starch–Gelatin Composite Films through the Incorporation of Zinc Oxide Nanoparticles

**DOI:** 10.3390/polym14122505

**Published:** 2022-06-20

**Authors:** Wantida Homthawornchoo, Pimonpan Kaewprachu, Suttiporn Pinijsuwan, Orapan Romruen, Saroat Rawdkuen

**Affiliations:** 1 Innovative Food Packaging and Biomaterials Unit, School of Agro-Industry, Mae Fah Luang University, Muang, Chiang Rai 57100, Thailand; suttiporn.pin@mfu.ac.th; 2 Food Science and Technology Program, School of Agro-Industry, Mae Fah Luang University, Chiang Rai 57100, Thailand; orapan.rom13@lamduan.mfu.ac.th; 3 College of Maritime Studies and Management, Chiang Mai University, Samut Sakhon 74000, Thailand; pimonpan.k@cmu.ac.th; 4 Cluster of Innovative Food and Agro-Industry, Chiang Mai University, Chiang Mai 50100, Thailand

**Keywords:** active film, *Centella asiatica* L. extract, nanocomposite, rice starch, uv barrier, zinc oxide nanoparticles

## Abstract

The effects of zinc oxide nanoparticles (ZnONPs) on the properties of rice starch–gelatin (RS–G) films were investigated. ZnONPs were synthesized by a green method utilizing Asiatic pennywort (*Centella asiatica* L.) extract. The ZnONPs were rod-shaped, with sizes ranging from 100–300 nm. An increase in the concentration of ZnONPs significantly (*p* < 0.05) increased the thickness (0.050–0.070 mm), tensile strength (3.49–4.63 MPa), water vapor permeability (5.52–7.45 × 10^−11^ g m/m^2^ s Pa), and thermal stability of the RS–G–ZnONPs nanocomposite films. On the other hand, elongation at break (92.20–37.68%) and film solubility (67.84–30.36%) were significantly lower (*p* < 0.05) than that of the control RS–G film (0% ZnONPs). Moreover, the addition of ZnONPs strongly affected the film appearance, color, transmission, and transparency. The ZnONPs had a profound effect on the UV-light barrier improvement of the RS–G film. The crystalline structure of the ZnONPs was observed in the fabricated nanocomposite films using X-ray diffraction analysis. Furthermore, the RS–G–ZnONPs nanocomposite films exhibited strong antimicrobial activity against all tested bacterial strains (*Staphylococcus aureus* TISTR 746, *Bacillus cereus* TISTR 687, *Escherichia coli* TISTR 527, *Salmonella Typhimurium* TISTR 1470) and antifungal activity toward *Aspergillus niger*. According to these findings, RS–G–ZnONPs nanocomposite film possesses a potential application as an active packaging: antimicrobial or UV protective.

## 1. Introduction

Recently, there has been an increased interest in developing biodegradable films owing to the environmental and marine life impacts. It has been reported that about 30% of all used packaging has not been appropriately discarded, causing the accumulation of packaging waste in the world’s lands and oceans [1]. Therefore, films derived from eco-friendly materials have gained attention in recent years as they have the potential to reduce or replace petroleum-based packaging. These natural polymers, such as polysaccharides (chitosan, carrageenan, cellulose, and starch), proteins (gelatin, soy protein, zein, and whey protein), and lipids (beeswax and fatty acids), are the potential raw materials used to produce biodegradable packaging films.

Rice starch is a polysaccharide extensively produced worldwide [2]. Approximately 90% (in dry weight) of the rice grain is its starch, while 6.5% and 0.8% are proteins and lipids, respectively [3]. Its renewability, biocompatibility, safety, and low cost make rice starch a potential raw material for producing polysaccharide-based packaging film. However, ricestarch-based films are rigid, exhibiting poor mechanical [4] and water barrier properties. In addition, the hydrophilicity and retrogradation mechanism in the rice-starch-based films, which decreases moisture permeability, results in inferior mechanical properties [5]. Incorporating other excellent film-forming biomaterials such as gelatin into the starch-based films enhanced the appearance and mechanical properties of the films [6,7,8].

Zinc oxide nanoparticles (ZnONPs) have attracted attention as reinforcing fillers in bio-nanocomposite materials for food applications as they are generally recognized as safe (GRAS) [9,10]. It also possesses excellent thermo-mechanical, outstanding UV barrier, and antimicrobial properties [11,12]. Generally, ZnONPs can be synthesized using physical, chemical, and biological (green) methods [13]. However, these days the green biosynthesis method, using plants, fungus, bacteria, and algae, has been extensively implemented to synthesize ZnONPs [14,15,16]. The application of the green methods is safe, simple, cost-effective, and environmentally friendly compared to the chemical and physical counterparts. The ZnONPs have been applied as a coating and reinforcing filler on variously based biopolymers such as polylactic acid (PLA) [17], chitosan-carboxymethyl cellulose [18], gelatin [19], and gelatin/cellulose nanofiber [20]. However, to the best of our knowledge, the information regarding the effect of the green synthesized ZnONPs on the properties of rice starch–gelatin bio-composite films remains scarce.

In this study, the ZnONPs were biosynthesized using Asiatic pennywort (*Centella asiatica* L.) extract. The Asiatic pennywort, a medicinal plant of the Apiaceae family, contains the main phytochemicals (i.e., tannins, flavonoids, glycosides, saponins, alkaloids, phenols, and terpenoids) that showed good antioxidant and antimicrobial activities [21]. The extract of Asiatic pennywort was used as reducing and capping (stabilizing) agents for ZnONPs biosynthesis [22,23]. The effects of green synthesized ZnONPs on the physicochemical, mechanical, barrier, thermal, and antimicrobial properties of the rice starch–gelatin-ZnONPs (RS–G–ZnONPs) nanocomposite films were subsequently investigated.

## 2. Materials and Methods

### 2.1. Materials and Microbials

Asiatic pennywort (*Centella asiatica* L.) leaves were obtained from a local market in Chiang Rai, Thailand. Rice starch was purchased from Cho Heng Rice Co., Ltd., Bangkok, Thailand. The commercial gelatin and zinc acetate dihydrate were purchased from Ajax Finechem (New South Wales, Australia). All other reagents used were of analytical grade.

*Staphylococcus aureus* TISTR 746, *Bacillus cereus* TISTR 687, *Escherichia coli* TISTR 527, *Salmonella* Typhimurium TISTR 1470, *Aspergillus niger*, and *Colletotrichum alatae* were obtained from the Biological Laboratory, Scientific and Technological Instruments Center, Mae Fah Luang University, Chiang Rai, Thailand.

### 2.2. Preparation of Asiatic Pennywort Extract and ZnONPs

Fresh leaves of Asiatic pennywort were washed in running tap water, followed by being dried at 50 °C overnight and ground into powder. The powder was passed through a sieve of 250 μm (60 mesh) to remove larger particles. In preparation of the Asiatic pennywort extract, 1 g of dried Asiatic pennywort leaf powder was mixed with 100 mL of distilled water. The mixture was incubated at 60 °C for 20 min, cooled down to room temperature, filtered through Whatman No.1 filter paper. The collected Asiatic pennywort extract was stored at 4 °C for further experiments.

A total of 50 milliliters of 1 M Zinc acetate were mixed with 50 mL of 2 M NaOH to synthesize ZnONPs. Then, 1 mL of Asiatic pennywort extract was added to the mixture and stirred continuously at room temperature for 2 h. After centrifugation at 10,000 rpm for 5 min, the pellet was washed with distilled water 3 times and then dried at 60 °C overnight [24]. Finally, the shape and size of the ZnONPs were evaluated using transmission electron microscopy (TEM) (Tecnai 12, Philips, Amsterdam, The Netherlands).

### 2.3. Preparation of Rice Starch–Gelatin Films Incorporated with ZnONPs

To prepare a ZnONP solution, ZnONPs at different concentrations (0, 0.5, 1, 2, and 3%, *w*/*v*) were mixed with distilled water. The designated concentration of ZnONPs was pre-determined based on the inhibition zone and minimum inhibitory concentration (MIC) via the disc diffusion method (data not included). The MIC of some microorganisms (i.e., *E. coli*, and *S. aureus*) agreed with the work of Naseer et al. [25] The mixture was stirred at 60 °C for 1 h and sonicated in an ultrasonic bath (Sonorex digitec, DT255H, Bandeline electronic, Berlin, Germany) for another 30 min to obtain a homogeneous mixture [26]. Then, rice starch–gelatin (RS–G) powder (3:1 ratio, *w*/*w*) and glycerol (30%, *w*/*w* with respect to the rice starch–gelatin content) were added to the ZnONP solution. The bio-nanocomposite film-forming solution (FFS) was heated and stirred continuously at 85 °C for 1 h, then cooled to 40 °C. The obtained FFS (4 ± 0.01 g) was then cast onto a rimmed silicone resin plate (50 mm × 50 mm) and left at room temperature for 24 h to evaporate the water content. The prepared nanocomposite films were dried in a dry cabinet (AH-80, Patron, San Francisco, CA, USA) at 25 ± 0.5 °C and 50 ± 5% relative humidity (RH) for 24 h. The control film was prepared by the same casting procedure but without any ZnONPs.

### 2.4. Film Properties Determinations

The bio-nanocomposite films were conditioned at 25 ± 0.5 °C and 50 ± 5% RH for 48 h before film characterization. For scanning electron microscopy (SEM), X-ray diffraction spectroscopy (XRD), Fourier transform infrared spectrometry (FT-IR), and thermo-gravimetric analysis (TGA), the films were dried in a desiccator containing dried silica gel at room temperature for two weeks to remove the excess water.

#### 2.4.1. Film Thickness

A digital thickness gauge (Mitutoyo, Tokyo, Japan) was used to determine film thickness. Six random locations were measured on each film sample. Five film specimens were used for each treatment.

#### 2.4.2. Mechanical Properties

Tensile strength (TS) and elongation at break (EAB) were analyzed using a Universal Testing Machine (Lloyd Instrument). The films were cut into the 20 mm × 50 mm specimens. The initial grip length was 30 mm, and the cross-head speed was 30 mm/min. Ten film specimens were tested for each film treatment.

#### 2.4.3. Film Appearance, Color, Optical Properties, and Morphology

The appearance of all film treatments was examined by using a digital camera (Fujifilm Finepix S4900, Fujifilm Thailand Co., Ltd., Bangkok, Thailand). A Color Quest XE (Hunter Lab) was used to assess the L* (lightness), a* (redness/greenness), and b* (yellowness/blueness) values of the prepared films. Color determination was conducted on five films per treatment. The total color difference (ΔE*) was calculated as follows Equation (1):

ΔE* = [(ΔL*)^2^ + (Δa*)^2^ + (Δb*)^2^]^0.5^ (1)

where ΔL*, Δa*, and Δb* are the differences in color value between e standard color plate and the film samples.

A UV-Vis spectrophotometer (Libra S22; Biochrom Ltd., Cambridge, UK) was used to evaluate light transmission in UV and visible range (200–800 nm) [27]. First, a film sample (40 mm × 40 mm) was placed into the spectrophotometer cell, and the transmission value was recorded at a wavelength of 600 nm. The transparency values of films were then calculated using the following Equation (2) [28]:

Transparency = log *T*_600_/*x* (2)

where *T*_600_ and *x* are the transmittance (%) at 600 nm and film thickness (mm), respectively.

Film morphology was analyzed by SEM (LE01450VP). The tests were carried out at magnifications of 5000× (surface) and 2000× (cross-section) with an acceleration voltage of 10 kV.

#### 2.4.4. Moisture Content and Film Solubility

Moisture content was examined following the Association of Official Analytical Chemists standard methods [29]. A film sample (20 mm × 20 mm) was dried at 105 °C for 24 h and then weighed. Each treatment was performed in triplicates.

The film solubility test was conducted in triplicates for each treatment according to the method described in [27]. Film solubility was calculated as the weight difference between the initial dry matter and the dried undissolved debris. The film solubility was expressed as a percentage of the total weight.

#### 2.4.5. Water Vapor Permeability

Water vapor permeability (WVP) measurement was conducted in accordance with a modified ASTM method [30]. A film sample was sealed into a WVP cup containing silica gel (0% RH). The cup was placed in a dry cabinet (AH-80, Patron) at 25 °C and 50% RH. Each cup was weighed every hour for 8 h. The WVP of the film was expressed as g m/m^2^ s Pa. The WVP test was carried out in triplicates for each film treatment.

#### 2.4.6. FT-IR Spectroscopy Analysis

An FT-IR Spectrum GX (PerkinElmer, Waltham, MA, USA) was used to analyze the FT-IR spectra of the films. Each film treatment was examined in triplicates at 25 °C using the spectrum range of 4000 to 650 cm^−1^ with 64 scans and a resolution of 4 cm^−1^ [27].

#### 2.4.7. XRD Analysis

The crystalline structures of film samples were analyzed using an X-ray diffractometer (X’Pert Pro MPD, Philips) with Cu Kα radiation (k = 0.154 nm) in a 2θ range between 20° and 80°.

#### 2.4.8. TGA Analysis

A thermo-gravimetric analyzer (Model 851e, Mettler Toledo, Columbus, OH, USA) was used to analyze the thermal stability of the films. Each film sample (10 mg) was put into a sample pan. The temperature was varied from 25 to 700 °C at 10 °C/min under a nitrogen atmosphere (20 mL/min).

#### 2.4.9. Antimicrobial Properties

A film sample (10-mm diameter) was exposed to UV light for 30 min. Subsequently, it was placed on a Muller–Hinton (MH) agar surface, inoculating with *S. aureus*, *B. cereus*, *E. coli*, *S. Typhimurium*, *A. niger*, and *C. alatae* [31]. The plates were then incubated at 25 °C and 37 °C for 18–24 h for fungal and bacterial testing, respectively. In addition, the inhibition zones around the film discs were measured. Experiments were carried out in triplicates. Ampicillin (10 μg/disc), streptomycin (10 μg/disc), and nystatin (100 units/disc) were used as antibiotics for the tested microorganisms.

### 2.5. Statistical Analysis

Data were expressed as mean ± standard deviation. In addition, the data were subjected to analysis of variance, and the differences between means were carried out by Duncan’s Multiple Range Tests. Statistical analysis was performed using the SPSS package (SPSS Inc., Chicago, IL, USA).

## 3. Results and Discussion

### 3.1. The Characterization of ZnONPs

**Figure 1:**
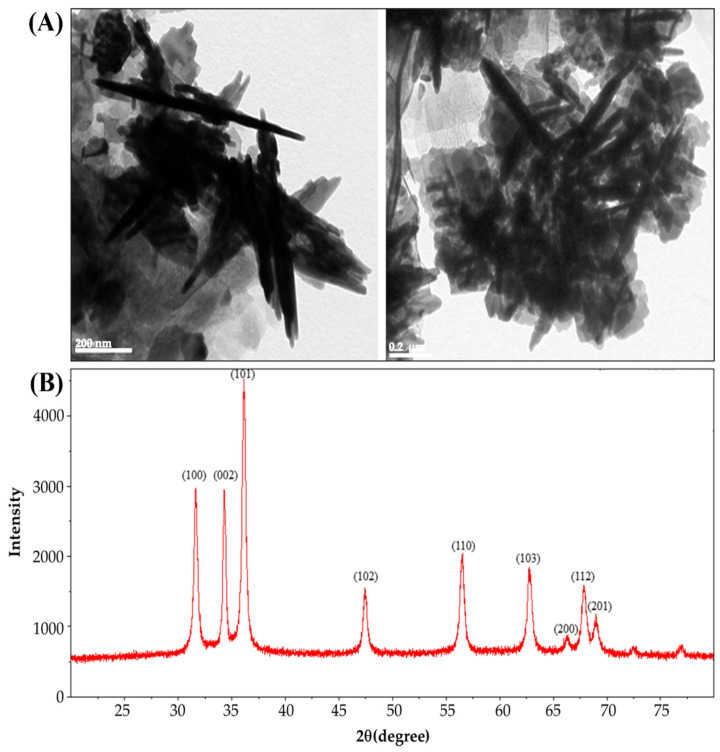
TEM (**A**) and XRD patterns, (**B**) of ZnONPs synthesized from Asiatic pennywort (*Centella asiatica* L.) extract.

The TEM images demonstrate (Figure 1A) that the green synthesized ZnONPs were rod-shaped with sizes ranging from 100 to 300 nm. Pure ZnONPs were confirmed by the presence of an XRD pattern. It was found that the XRD spectra of the obtained ZnONPs exhibited major characteristic diffraction peaks at 2θ = 31.6, 34.2, 36.1, 47.4, 56.5, 62.7, 66.2, 67.8, and 69.2, corresponding to (100), (002), (101), (102), (110), (103), (200), (112), and (201) planes of zinc oxide nanoparticles, in respective order (Figure 1B). The diffraction peaks were observed due to a hexagonal wurtzite structure of zinc oxide. Our results agreed with that reported by Bhatte et al. [32], Bhuyan et al. [33], and Naseer et al. [25].

### 3.2. Properties of RS–G–ZnONPs Nanocomposite Films

#### 3.2.1. Film Thickness

The thicknesses of the rice starch–gelatin films at different concentrations of ZnONPs are shown in Table 1. It was found that the control rice starch–gelatin (RS–G) film (without ZnONPs) was the thinnest. The addition of ZnONPs (i.e., 0.5–3%, *w*/*v*) led to a significant increase in the thickness (*p* < 0.05) of the films. At the highest concentration of ZnONP incorporation (i.e., 3%, *w*/*v*), the developed film showed the greatest increase in film thickness. An increase in the solid content and the less compact structure due to ZnONPs might be responsible for the thickness increases in the developed film, resulting in a thicker film [34,35]. The thickness increase alongside the incorporation of ZnONPs was similarly observed in other biopolymer-based films, such as gelatin film [36].

#### 3.2.2. Mechanical Properties

The mechanical properties of RS–G films with ZnONPs are tabulated in Table 1. The TS and EAB were affected by the addition of ZnONPs. The control RS–G film (without ZnONPs) had a TS and EAB of 3.49 MPa and 92.20%, respectively. After the inclusion of ZnONPs into the RS–G matrix, the TS of composite films increased from 3.49 to 4.63 MPa, while the EAB significantly decreased from 92.20 to 37.68% (*p* < 0.05). The increase in TS is presumably due to the strong molecular interactions between ZnONPs and the RS–G matrix [34]. These results could indicate that the ZnONPs and the biopolymer matrix have high compatibility, resulting in rigid films. However, these interactions could restrict the mobility of polymer chains which decreases the EAB. Furthermore, the water content in the starch matrix could also act as a plasticizer that contributes to the flexibility of the resulting films [37]. Thus, the decrease in water content as a result of the incorporation of ZnONPs may reduce the flexibility of the starch-based films. Additionally, a large surface area of ZnONPs could also serve as nanofillers reinforcing the film matrix through interfacial interactions [38]. A similar finding of increasing the TS and reducing EAB has been observed in the zinc oxide nanorods-gelatin-based film [36] and ZnONPs-*Gracilaria vermiculophylla* extract films [39].

#### 3.2.3. Film Appearance, Color, Optical Properties, and Morphology

**Figure 2:**
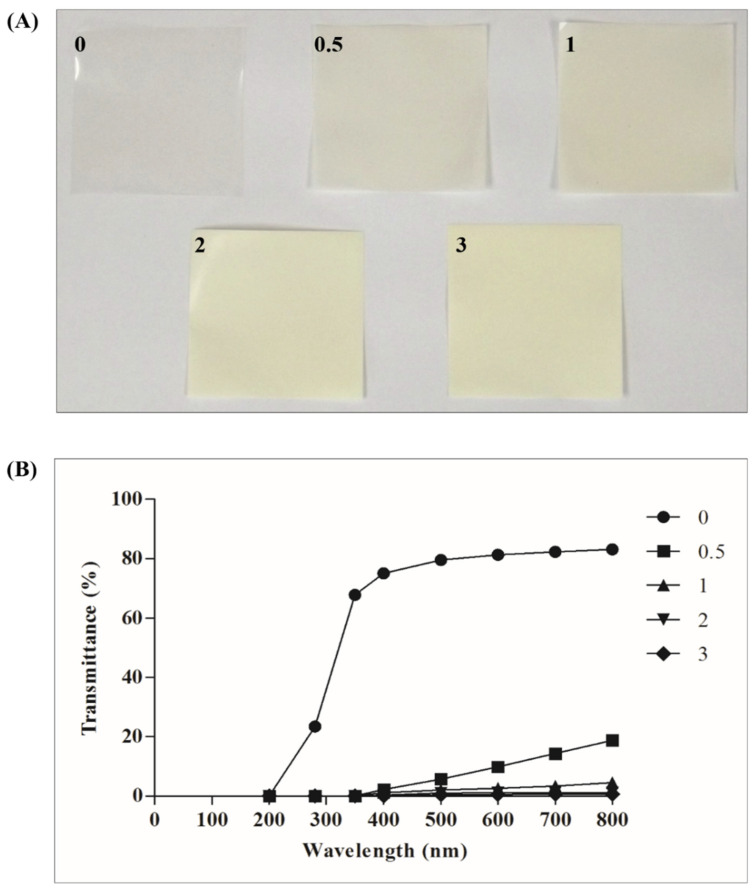
Film appearance and color values (**A**) (0% ZnONPs, L* = 86.10 ± 0.10, a* = −1.30 ± 0.06, b* = 2.48 ± 0.08, ∆E* = 8.64 ± 0.08; 0.5% ZnONPs, L* = 86.56 ± 0.15, a* = −2.68 ± 0.03, b* = 5.40 ± 0.39, ∆E* = 9.53 ± 0.31; 1% ZnONPs, L* = 87.10 ± 0.07, a* = −3.00 ± 0.13, b* = 7.14 ± 0.58, ∆E* = 10.22 ± 0.44; 2% ZnONPs, L* = 88.04 ± 0.15, a* = −1.62 ± 0.08, b* = 10.74 ± 0.27, ∆E* = 12.25 ± 0.24; 3% ZnONPs, L* = 89.06 ± 0.17, a* = −1.46 ± 0.03, b* = 12.16 ± 0.34, ∆E* = 13.01 ± 0.36), and light transmittance (**B**) of RS–G films incorporated with different concentrations of ZnONPs. The numbers designate the ZnONPs concentrations (%, *w*/*v*).

Film appearance and color attributes of the RS–G films at various concentrations of ZnONPs are presented in Figure 2A. The control films were colorless and transparent. The L*, a*, b*, and ΔE* values of the control film were 86.10, −1.30, 2.48, and 8.64, respectively. As the concentration of ZnONPs increased from 0.5% to 3% (*w*/*v*), the RS–G films became more turbid and milkier. These changes agree with the increasing tendency in the color values of L* (86.56–89.06), a* ((−2.68)–(−1.46)), b* (5.40–12.16), and the total color change (ΔE*) (9.53–13.01) of the RS–G–ZnONPs nanocomposite films being proportional to the ZnONPs concentration. A similar whitening effect has also been reported in linear low-density polyethylene (LLDPE)-ZnONPs composite films [40].

The optical properties of films were expressed by light transmission and transparency. The light transmission values of the RS–G–ZnONPs nanocomposite films at the wavelength between 200 and 800 nm are shown in Figure 2B. The UV-light transmission (200–280 nm) of the control RS–G composite films (without ZnONPs) varied between 0.06 and 67.80%. At high concentrations of ZnONPs (>0.5%, *w*/*v*), the RS–G–ZnONPs nanocomposite films did not show any UV-light transmission. Light transmission in the visible range (400–800 nm) of the control film (without ZnONPs) and the RS–G–ZnONPs nanocomposite films were in the range of (75.07–83.15) and (0.33–18.82)%, respectively. The results suggested that adding ZnONPs into RS–G composite films decreased UV- and visible-light transmission. This phenomenon was caused by light scattering due to ZnONPs present in the film matrix [41]. The UV- and visible-light-shielding effect were also found in other ZnONPs-added nanocomposite films, such as *Gracilaria vermiculophylla* [39], LLDPE [40], and gelatin [36] films. This finding would benefit the development of UV protective packaging films that could reduce oxidative deterioration in fatty foods.

The transparency of the RS–G–ZnONPs nanocomposite films is illustrated in Table 1. The transparency of the control RS–G composite film (without ZnONPs) was the highest. The addition of the ZnONPs into the RS–G bio-composite films significantly (*p* < 0.05) decreased the transparency of the resulting films. The opaquest film was the RS–G bio-composite film incorporating ZnONPs (3%, *w*/*v*). The transparency of the film was determined by ZnONP addition and the compatibility of the ZnONPs to the biopolymer base [42]. This RS–G–ZnONPs nanocomposite film might not be suitable for see-through food packaging to show the food’s appearance and color inside the package. However, these opaque films would be of great application as a UV-light barrier packaging film. A similar UV-shielding effect of ZnONPs has been observed in buckwheat starch films [38].

**Figure 3:**
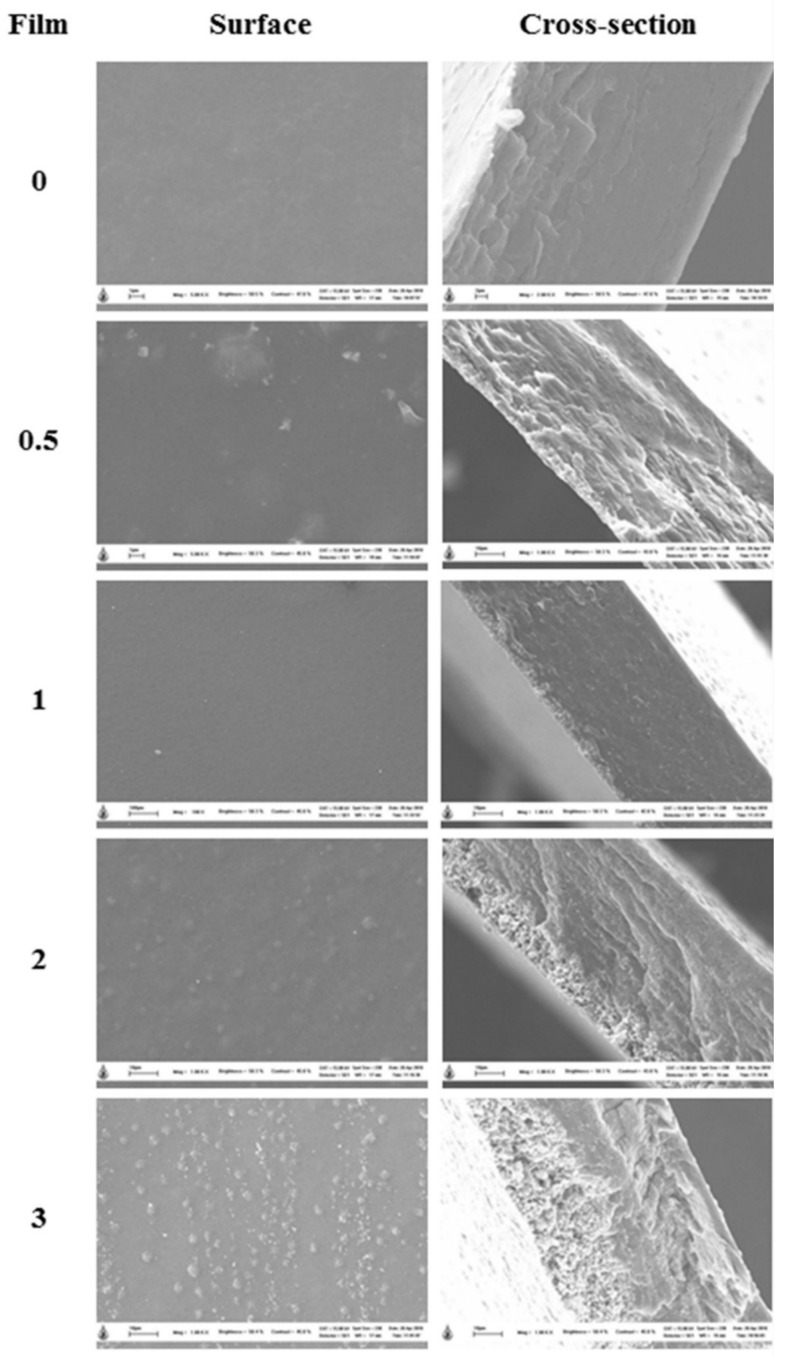
SEM micrographs of surface (magnification: 5000×) and cross-section (magnification: 2000×) of RS–G films containing different concentrations of ZnONPs. Numbers denote the ZnONPs concentrations (%, *w*/*v*).

The SEM-observed surface and cross-sectional morphology of different RS–G–ZnONPs nanocomposite films (0–3%, *w*/*v* ZnONPs) are shown in Figure 3. It was found that the control RS–G composite film (without ZnONPs) showed a smooth, homogenous, and compact surface and cross-sectional micrographs. A similar report of a smooth surface film was found in the native rice starch film [43]. In contrast, the RS–G–ZnONPs nanocomposite films exhibited a rough surface and cross-section morphology. The roughness of the films was proportional to the increasing concentration of ZnONPs in the films. The SEM surface micrographs showed that the ZnONPs evenly dispersed and protruded the RS–G composite films. The cross-sectional images indicate that the ZnONPs were self-aggregated in the matrix of the developed films, causing the heterogeneity of the film cross-sections. The results obtained were associated with the increase in the WVP and thickness values (Table 1) when incorporating the higher concentrations of ZnONPs in the nanocomposite films. The effects of ZnONPs on the alteration of film morphology have also been illustrated in the fish gelatin film [44], fish protein isolate/fish gelatin film [41], LLDPE film [40], and buckwheat starch film [38].

#### 3.2.4. Moisture Content and Film Solubility

The moisture contents and the film solubility of the RS–G films reinforced with ZnONPs at different concentrations are shown in Table 1. The developed films exhibited moisture contents ranging between 13.08 and 18.86%. The control film had the highest moisture content (18.86%). As the ZnONP concentration increased from 0 to 3%, *w*/*v*, the moisture content of RS–G–ZnONPs nanocomposite films decreased significantly (*p* < 0.05). The moisture content reduction in the nanocomposite film might result from nanoparticle addition, as it increased the hydrophobicity characteristic of the resulted films [45,46,47]. Furthermore, the increasing interaction of hydrogen bonding between nanoparticles and hydroxyl groups in the film matrix also limited the availability of the hydroxyl groups from bonding with water [37,48]. Consequently, the RS–G–ZnONPs nanocomposite films exhibited lower moisture content than the RS–G composite film (without ZnONPs). Additionally, the lower moisture content in the nanocomposite films was related to the mechanical properties of the film as the water is served as a plasticizer in the bio-composite film. Thus, decreasing moisture content resulted in the higher TS values and the lower EAB values of the developed films (Table 1). A similar effect of nanoparticle addition on the decreasing moisture contents in various biopolymer-based films has been reported in chitosan [47], soluble soybean polysaccharide [37], agar/banana powder [34], pectin [49], buckwheat starch [38].

The solubility of the RS–G–ZnONPs nanocomposite films varied between 30.36 and 67.84%. The RS–G-blend film had the highest film solubility. A significant decrease (*p* < 0.05) in the film solubility (63.23–30.36%) was observed when the concentration of ZnONPs was increased from 1 to 3% (*w*/*v*). The lower film solubility of the nanocomposite corresponded to the lower moisture contents due to the lower availability of the hydroxy groups to scavenge the water, as discussed earlier. Thus, the lowest film solubility was found at the highest ZnONP content in the RS–G–ZnONPs nanocomposite film (Table 1). Similar results have been discovered in other biopolymer-based films, for example, *Gracilaria vermiculophylla* [39], buckwheat starch [38], and corn starch [50].

#### 3.2.5. Water Vapor Permeability (WVP)

The WVP values of RS–G–ZnONPs nanocomposite films ranged from 5.52–7.45 × 10^−11^ g m/m^2^ s Pa and are illustrated in Table 1. The control rice starch–gelatin film (without ZnONPs) showed the lowest WVP value (5.52 × 10^−11^ g m/m^2^ s Pa), while films containing 3% (*w*/*v*) of ZnONPs exhibited the highest (7.45 × 10^−11^ g m/m^2^ s Pa) WVP value.

Generally, the nanoparticles’ inclusion reduced the WVP of the resulting nanocomposite film. This typical result was possibly due to the tortuous pathway for a water molecule to travel through the film and the limited hydrophilicity portions [20,51,52]. However, at a high concentration of nanoparticle addition (>1%, *w*/*v*), the increasing WVP of the developed films has been reported [39,53]. In this study, the increasing WVP values were significantly observed in the developed nanocomposite film with ZnONPs higher than 1%, *w*/*v*. The possible explanation for this phenomenon might be the high porosity (roughness) and the void space caused by ZnONPs aggregation in the film matrix at high ZnONPs concentrations [54]. Additionally, the disrupted structure and discontinuous complex biopolymer matrix caused by nanoparticle addition might also be responsible for the event [55]. A similar observation has been illustrated in *Gracilaria vermiculophylla* films incorporated with ZnONPs [39].

#### 3.2.6. FT-IR Spectroscopy

**Figure 4:**
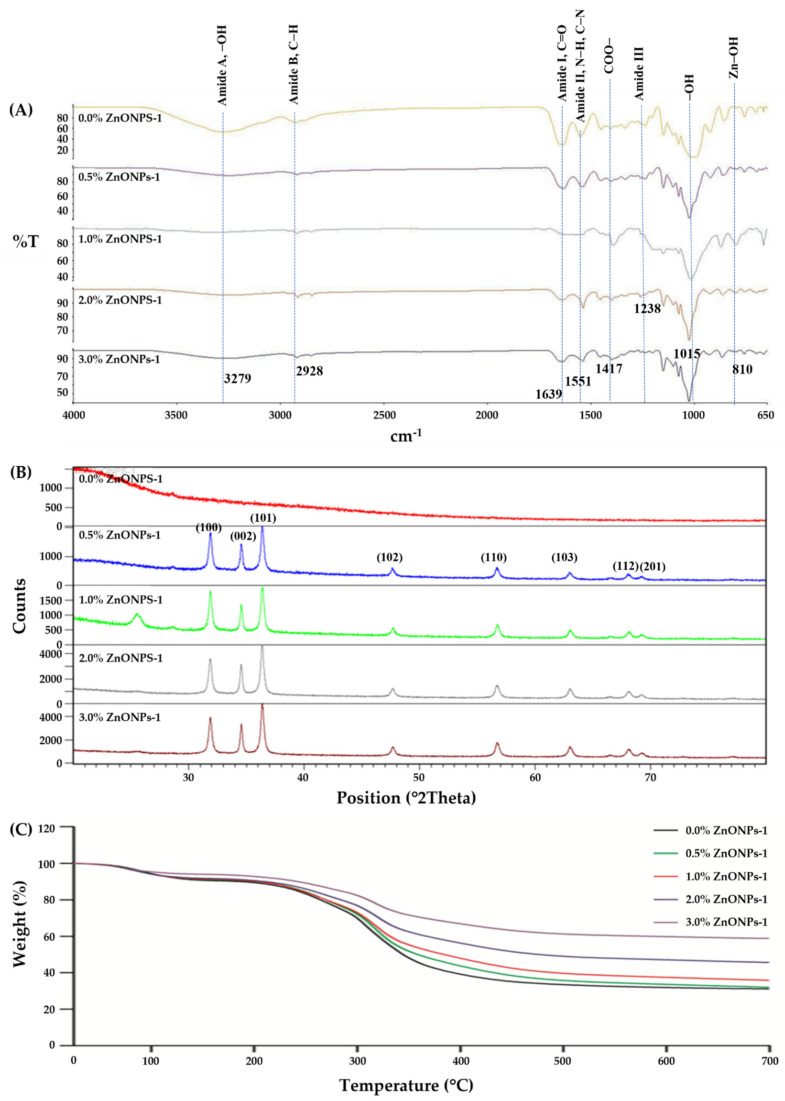
FT-IR spectra (**A**), XRD pattern (**B**), and TGA curves (**C**) of RS–G films incorporated with different concentrations of ZnONPs. Numbers denote the ZnONPs concentrations (%, *w*/*v*).

The interactions between rice starch–gelatin matrix and ZnONPs were monitored using FT-IR analysis. Figure 4A represents FT-IR spectra of the RS–G films incorporated with different ZnONP concentrations. The spectra of all nanocomposite films displayed the IR bands varying from 3500 to 650 cm^−1^. All RS–G films exhibited the typical characteristic peaks of rice starch [56], gelatin [41], and the RS–G composite [6] at 3278.98 cm^−1^ (amide-A, representing O–H stretching), 2927.68 cm^−1^ (amide-B, relating to C–H stretching), 1639.09 cm^−1^ (amide-I, exhibiting C=O stretching), 1551.47 cm^−1^ (amide-II, representing the deformation of N–H and C–N stretching), 1238.43 cm^−1^ (amide-III, displaying vibrations of C–N and N–H groups in plane). The presence of ZnONP was confirmed by the IR peak of Zn-OH group at 810 cm^−1^. This Zn-OH peak was also observed in the report of Doan Thi et al. [57] In addition, the IR peaks at 1015 cm^−1^ of all RS–G–ZnONPs designate the interaction of the O–H group of glycerol with the film structure [41]. The shift of this peak towards the higher wavenumber indicated some conformational changes in the functional group or complex forming with the larger functional groups [6,58]. The peak at 1417 cm^−1^ represents the symmetric stretching of the COO– group. The shift of this peak to a lower wavenumber was observed, indicating the weaker –OH groups and COO– group interaction [43] in the RS–G–ZnONPs matrix.

All nanocomposite films containing different ZnONP content did not differ in the vibrational wavenumber for amide-B, amide-I, amind-II and amide-III peaks, except for the amide-A peak. The slight shifts of the peak in the amide-A region from 3278.98 cm^−1^ (0% ZnONPs) [59] to a lower wavenumber (3250.61, 3229.96, 3241.25, and 3260.11 cm^−1^ for 0.5 to 3% ZnONPs (*w*/*v*), respectively) when adding ZnONPs into the RS–G composite films were observed. These IR changes in the amide-A region, indicating that the functional groups of the biopolymers adsorbed on the ZnONPs, were related with the interactions of the N–H group in gelatin with ZnONPs through hydrogen bonding [41,44]. This shift in IR bands of amide-A was associated with the changes in mechanical properties, namely the increase in the TS and decrease in EAB of the RS–G–ZnONPs composite films. Similar reports were found by Kaewprachu et al. [58] and Arfat et al. [41]

#### 3.2.7. XRD Results

XRD analysis was used to determine the crystalline structure of the RS–G film and their nanocomposite films. According to the report of Rouhi et al. [44], there was no XRD diffraction peaks observed in the fish gelatin film. In addition, according to Suriyatem et al. [43], the native rice starch film exhibited a semi-crystalline matrix with a Vh-type crystalline polymorphic structure at 13.8 and 21.9°. However, in this work, the XRD diffraction peak (Figure 4B) investigation found that no XRD patterns of the control RS–G composite films were observed at 0% ZnONPs. This phenomenon indicates an amorphous behavior of the control RS–G film, implying that a typical A-type crystalline polymorph of the native rice starch was degraded by the film preparation [43,60], possibly through the complex formation of amylopectin and gelatin. However, when the ZnONPs were incorporated into the RS–G composite films, prominent characteristic diffraction peaks at 31.6, 34.2, 36.1, 47.4, 56.5, 62.7, 67.8, and 69.2° of 2θ were observed, which corresponded to (100), (002), (101), (102), (110), (103), (112), and (201) planes of zinc oxide nanoparticles, respectively. At higher concentrations of ZnONPs, the aforementioned XRD diffraction peaks of the RS–G films showed stronger signals indicating a high portion of crystalline structure. Similar observations were also found in other biopolymer-based films with ZnONPs (i.e., cellulose [61], carrageenan [11], chitosan-carboxymethyl cellulose [18], fish gelatin [44] and fish protein isolate/fish skin gelatin [41]).

#### 3.2.8. TGA Results

The thermal stability of the RS–G–ZnONPs nanocomposite films was measured by using TGA. TGA thermograms of the developed films are shown in Figure 4C. The results showed two main steps of thermal degradations for the control film and the RS–G–ZnONPs nanocomposite films. The initial thermal degradation was observed between 80–95 °C with weight loss ranging from 5.98 to 9.69%, indicating the loss of water content from the films. The second thermal degradation was revealed at approximately 310–320 °C, which can be attributed to the thermal decomposition of polymers [62]. The heat resistance of the RS–G–ZnONPs nanocomposite films increased with ZnONPs content. After the final thermal decomposition, the percentage of residue at around 700 °C for the control rice starch–gelatin film and nanocomposite films added with 0.5–3% (*w*/*v*) of ZnONPs were 27.95, 32.50, 34.79, 45.26, and 56.93%, respectively. Thus, the inclusion of ZnONPs into the RS–G film increased the thermal stability of the resulting films. This phenomenon might be attributed to the properties of the ZnONPs, such as heat insulation, the enhancement of polymer chain interaction, and the escape of volatile compounds blocking [63]. Similar findings have been observed in ZnONPs-fabricated carrageenan films [11], and alginate films containing halloysite nanotubes and ZnONPs [17].

#### 3.2.9. Antimicrobial Activity

**Figure 5:**
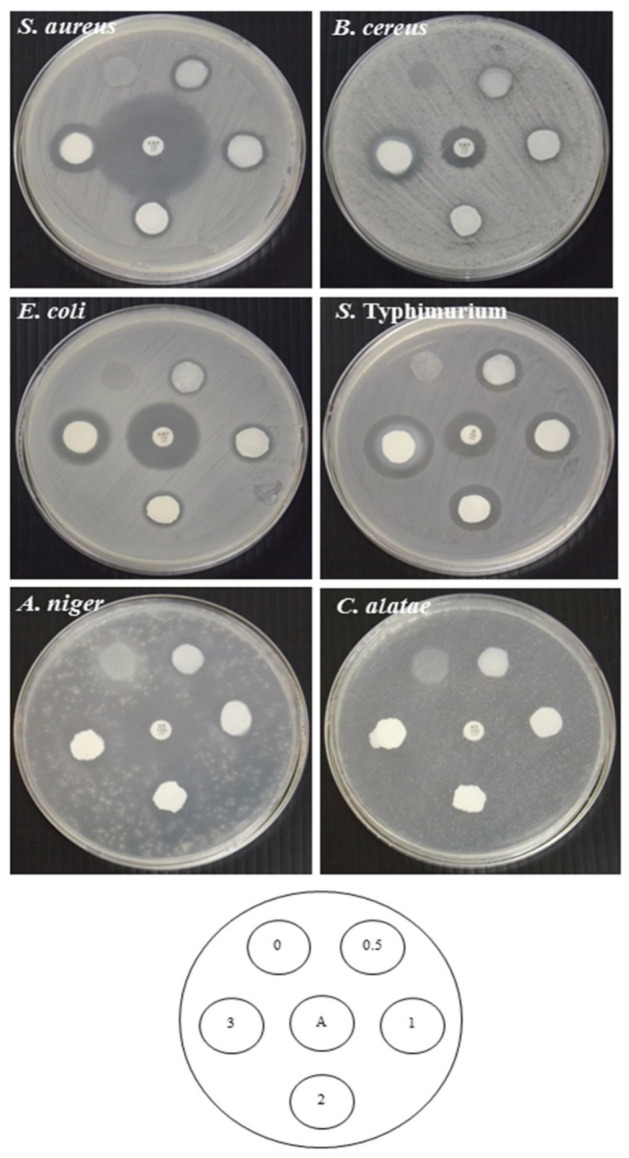
Antimicrobial properties of RS–G films containing different concentrations of ZnONPs. The numbers designate of ZnONPs concentrations (%, *w*/*v*). A: ampicillin (10 μg/disc), streptomycin (10 μg/disc), and nystatin (100 units/disc).

Antimicrobial activity of RS–G films with ZnONPs was tested against Gram-positive bacteria (*S. aureus* and *B. cereus*), Gram-negative bacteria (*E. coli* and *S. Typhimurium*), and fungi (*A. niger* and *C. alatae*) by the disc diffusion method in comparison with the control RS–G film (Figure 5). All RS–G–ZnONPs nanocomposite films showed strong antibacterial effects on all bacterial strains tested. The antibacterial activity of ZnONPs against *E. coli* and *S. aureus* were also found in the report of Doan Thi et al. [57], Naseer et al. [25]. The antifungal ability of all RS–G–ZnOPNS films showed good activity on *A. niger*. The inhibition of *A. niger* was also observed in Naseer et al. [25]. However, there was no observable clear zone on *C. alatae*. The decrease in reactive oxygen species (ROS) (i.e., H_2_O_2_, OH^−^, or O_2_-radicals), generated from the interactions of ZnONPs with water [57,64,65], was possibly responsible for this phenomenon. ROS affected the antibacterial [66] and antifungal abilities [65] of the ZnONPs-containing films. The reduction in antifungal inhibition might be due to the RS–G–ZnONPs films possessing less water binding ability when increasing the concentration of the ZnONPs in the films. The control RS–G film did not show any antimicrobial activity. An increase in the observable clear zone was proportional to the amount of incorporated ZnONPs. Baek and Song [39] also reported an increase in the antimicrobial effect of *Gracilaria vermiculophylla* films against *L. monocytogenes* and *S. Typhimurium* with the increase in the amount of ZnONPs. Jebel et al. [61] reported that the inclusion of ZnONPs enhanced the antimicrobial activity of the cellulose-based films against *S. aureus* and *E. coli*. The strong antimicrobial activity of ZnONPs was most likely due to disruption of the cell membrane of microorganisms caused by Zn^2^^+^ ions. The ZnONPs have been reported to mediate the generation of hydrogen peroxide (H_2_O_2_), a powerful oxidizing agent, which causes damage to the cell membrane [67]. According to this finding, the RS–G–ZnONPs nanocomposite films have the potential to be used as antimicrobial food packaging.

## 4. Conclusions

The effects of ZnONPs on the properties of the RS–G composite films were investigated. The results revealed that the inclusion of ZnONPs improved the UV-light barrier, thermal stability, tensile strength, and antimicrobial activity against all tested bacterial strains and *A. niger* fungi. In addition, the thickness and water vapor permeability of the developed films were also increased. On the other hand, the ZnONPs significantly decreased the transparency, EAB, moisture content, and film solubility compared to the control RS–G film (without ZnONPs). The presence of ZnONPs also changed the crystalline properties of the rice starch–gelatin films. Therefore, the RS–G–ZnONPs nanocomposite films have good antibacterial, heat-resistant, and UV-protective properties that are potentially useful for food packaging applications. However, at a high concentration of ZnONP incorporation, the opacity and elasticity of the RS–G–ZnONPs nanocomposite film might be compromised. Additionally, the antifungal properties of the RS–G–ZnONPs composite films to inhibit *C. alatae* and other pathogenic fungi should be further investigated at higher concentrations of ZnONPs for antifungal applications in the food and packaging industry.

## Figures and Tables

**Table 1:** Physicochemical, mechanical, and barrier properties of RS–G films reinforced with different concentrations of ZnONPs.

ZnONPs (% *w*/*v*)	Thickness (mm)	TS (MPa)	EAB (%)	Transparency	MC (%)	FS (%)	WVP (× 10^−^^11^ g m/m^2^ s Pa)
0	0.050 ± 0.002 ^d^	3.49 ± 0.31 ^c^	92.20 ± 9.74 ^a^	3.21 ± 0.01 ^a^	18.86 ± 1.85 ^a^	67.84 ± 1.02 ^a^	5.52 ± 0.25 ^b^
0.5	0.055 ± 0.002 ^c^	3.55 ± 0.18 ^c^	57.43 ± 3.43 ^b^	2.25 ± 0.06 ^b^	17.15 ± 0.69 ^ab^	63.23 ± 0.28 ^b^	5.84 ± 0.28 ^b^
1	0.060 ± 0.001 ^b^	3.66 ± 0.20 ^bc^	52.10 ± 8.55 ^b^	1.64 ± 0.02 ^c^	17.02 ± 1.15 ^ab^	48.07 ± 0.95 ^c^	6.15 ± 0.32 ^b^
2	0.069 ± 0.002 ^a^	3.80 ± 0.17 ^b^	43.75 ± 8.55 ^c^	1.22 ± 0.09 ^d^	15.02 ± 0.45 ^bc^	40.61 ± 2.02 ^d^	7.26 ± 0.49 ^a^
3	0.070 ± 0.001 ^a^	4.63 ± 0.28 ^a^	37.68 ± 2.58 ^d^	0.91 ± 0.08 ^e^	13.08 ± 1.02 ^c^	30.36 ± 1.72 ^e^	7.45 ± 0.25 ^a^

Values are given as mean ± SD from *n* = 5 determination for thickness; *n* = 10 for determinations of TS and EAB; *n* = 3 for determinations of MC, FS, and WVP. Different superscripts (^a–e^) in each column are significantly different (*p* < 0.05). ZnONPs: zinc oxide nanoparticles; TS: tensile strength; EAB: elongation at break; MC: moisture content; FS: film solubility; WVP: water vapor permeability.

## Data Availability

The data presented in this study are available on request from the corresponding author.
